# Who benefits from multidisciplinary care in functional somatic disorders? Identifying cost-effective patient selection through diagnostic classification groups

**DOI:** 10.1186/s13584-026-00750-7

**Published:** 2026-03-18

**Authors:** Oded Hammerman, Alon Rasooly, Dan Greenberg, Talma Kushnir, Erez Yaakobi, Yacov Ezra

**Affiliations:** 1 Department of Neurology, Soroka Medical Center, Be’er-Sheva, Israel; 2 Department of Health Policy and Management, School of Public Health, Faculty of Health Sciences, Ben-Gurion University of the Negev, Be’er-Sheva, Israel; 3 Department of Psychology and Adelson School of Medicine, Ariel University, Ariel, Israel; 4 School of Public Health, Faculty of Health Sciences, Ben-Gurion University of the Negev, Be’er-Sheva, Israel; 5 Faculty of Business Administration, Ono Academic College, Kiryat-Ono, Israel

**Keywords:** Functional somatic disorders, Medically unexplained symptoms, Healthcare utilization, Healthcare costs, Multidisciplinary care

## Abstract

**Background:**

Functional Somatic Disorders (FSD) significantly impact patients’ quality of life while placing a substantial economic burden on healthcare systems. Identifying which patients achieve reductions in healthcare costs through multidisciplinary care remains crucial for optimizing resource allocation.

**Methods:**

A retrospective analysis examined healthcare utilization and costs during the year preceding and the year following patients’ initiation of treatment at a specialized, multidisciplinary clinic in Israel. The clinic combined medical care with mind-body therapies - including cognitive behavioral therapy, hypnotherapy, and physiotherapy. Using baseline characteristics, patients were divided into Diagnostic Classification Groups (DCG). A multivariable analysis was performed to ascertain the impact of DCG on annual, average changes in healthcare utilization and costs after treatment.

**Results:**

Data from *N* = 685 patients were analyzed. Reduced healthcare utilization and costs were observed among approximately 56% of the population receiving multidisciplinary care. Mean annual cost reductions were 1,367 ILS per patient. Specifically, significant reductions were observed in hospitalizations (-1,723 ILS) and diagnostic procedures (-495 ILS). Patients with simple FSD and stress-exacerbated diseases showed significant cost reductions (*P* < 0.05), while those with organic disease or difficult FSD did not demonstrate significant changes. Analysis by DCG revealed that baseline diagnostic classification significantly predicted cost reduction.

**Conclusion:**

Healthcare utilization and costs were reduced among FSD patients receiving multidisciplinary treatment, with outcomes varying by diagnostic classification. Baseline diagnostic classification reliably predicted treatment cost-effectiveness, underlining the economic value of collaborative care for patients with simple functional disorders and stress-exacerbated diseases. These diagnostic patterns provide healthcare policymakers with selection criteria for multidisciplinary programs and inform the development of tailored interventions.

**Supplementary Information:**

The online version contains supplementary material available at 10.1186/s13584-026-00750-7.

## Introduction

Functional Somatic Disorders (FSD) refer to persistent and distressing physical symptoms arising from complex interactions between bodily and brain processes. [1] These conditions – commonly manifesting as chronic pain, irritable bowel syndrome, tension headaches, and fibromyalgia, among others – are highly prevalent and associated with substantial impairment and elevated rates of anxiety and depression. [2] FSD are extremely common, with one-third of consultations in primary care and specialist settings estimated to be due to these conditions [3, 4]. FSD also account for a considerable share of healthcare use, with patients incurring significantly higher outpatient and inpatient costs than those without such disorders [5].

Clinical guidelines recommend multidisciplinary care for FSD, integrating medical management with non-pharmacological approaches, including mind–body therapies (MBT) such as hypnotherapy, cognitive behavioral therapy, mindfulness meditation, and pain reprocessing therapy [6, 7]. Multidisciplinary, mind-body interventions aim to reduce symptom burden, improve functioning, and potentially lower healthcare utilization and costs (HUC) [8, 9]. In multidisciplinary settings, treatment is provided through a stepped-care approach, in which the most severely affected patients receive the most comprehensive level of care [10] Despite evidence supporting their clinical value, multidisciplinary services remain limited in routine practice [11].

In Israel, access to multidisciplinary care is particularly constrained. Although recent mental health reforms expanded insurance coverage for psychological services, no formal policy exists for implementing multidisciplinary FSD treatment, and specialized services remain scarce [12, 13]. As a result, many clinicians lack appropriate referral options, and patients often face barriers to receiving MBT-based care. Currently, only a handful of specialized services exist nationwide. The Israel Pain Association lists 21 pain clinics, but their multidisciplinary nature remains unclear, [14] while the Israel Psychosomatic Society primarily lists private services not integrated with the public system [15]. Consequently, most physicians treating FSD face personal and systemic obstacles, including a lack of competent referral services due to insufficient resource allocation toward MBT-specialized treatment centers [16, 17]. One critical consideration for policy change is the economic viability of such treatments.

While several studies have examined multidisciplinary and MBT interventions for FSD, findings on their economic impact are inconsistent [18–21]. Few investigations have directly assessed changes in healthcare utilization, and even fewer have examined which patient characteristics predict cost reductions. Economic reviews suggest that treatment effectiveness likely depends on multiple factors, including comorbid conditions, specific diagnoses, and the type of intervention [22, 23]. This variability in economic outcomes highlights a critical question in the field. As noted by Stahl et al.,^24^ while MBTs can reduce healthcare utilization, we lack reliable methods to identify which patients will benefit most. This gap limits healthcare systems’ ability to allocate intensive treatments efficiently.

This study addresses these challenges by examining changes in healthcare utilization and costs among patients with FSD receiving multidisciplinary care that includes MBT. The objectives of this study were (1) to investigate changes in healthcare utilization and costs for patients with FSD receiving multidisciplinary care, including MBT, and (2) to determine whether patients’ baseline diagnostic factors could predict these changes. This novel study goes beyond evaluating the extent to which multidisciplinary care reduces healthcare costs by identifying which patients are most likely to benefit from it. Identifying which patients benefit most may support more targeted use of resource-intensive services and inform future policy development.

## Methods

### Study population

This study was based on a retrospective, pre- and post-treatment database analysis that explored the HUC of all patients presenting for care at the Functional Neurology Clinic (FNC) at Soroka Medical Center from 2011 to 2016. Electronic Medical Records (EMRs) of patients aged 18 years or older were obtained from Clalit Health Services (CHS), Israel’s largest health maintenance organization. The FNC is a tertiary, outpatient clinic established in 2011 at Soroka Medical Center in Be’er Sheva, Israel. The clinic’s treatment team comprises a neurologist, a psychiatrist, a physical therapist, a social worker, five health psychologists, and administrative staff. Treatment was provided through a stepped-care approach, [10] combining standard medical investigation and disease management with mind-body therapies (such as hypnotherapy, biofeedback, and mindfulness meditation), cognitive behavioral therapy, and physiotherapy.

As the primary objective of establishing the specialized clinic was to treat individuals with FSDs, most patients were referred to the FNC for this reason and received multidisciplinary care that specifically included MBT. A number of patients with organic neurological diseases (e.g., epilepsy, CVA, etc.) were also referred and received standard medical care. All FNC patients presented with neurological symptoms and were initially assessed by a neurologist. Following the assessment, patients were referred to additional team members based on the medical evaluation. Psychological care and physiotherapy were the second-tier treatments. Psychiatric consultation was reserved for those patients in need of psychopharmacological treatment or other types of psychiatric care. Patients in need of socioeconomic assistance (e.g., help receiving social or disability benefits) were referred to the social worker. Clinical decisions were made within a multidisciplinary framework at weekly staff meetings. A number of group therapy interventions were also provided weekly, including a mindfulness-based cognitive therapy (MBCT) group and a “walking group” in which a psychologist and a physiotherapist collaborated to encourage physical activity among patients with chronic pain.

## Study procedure

To determine who is most likely to benefit from multidisciplinary treatment, patients were stratified according to the neurologist’s diagnosis at the FNC upon arrival, which was then consolidated with medical information from the EMR. This information included the presence of severe organic diseases (e.g., cancer, renal failure, congestive heart failure, cirrhosis of the liver, cerebrovascular accident). Less severe organic diagnoses (e.g., diabetes without severe complications, hypothyroidism) were not used as stratification criteria because they were common and therefore did not distinguish between groups, nor did they directly relate to patients’ FSD or to care received at the FNC. Through this process, five diagnostic classification groups (DCGs) were delineated and could be identified using baseline diagnostic criteria in the EMR.

**Fig. 1:**
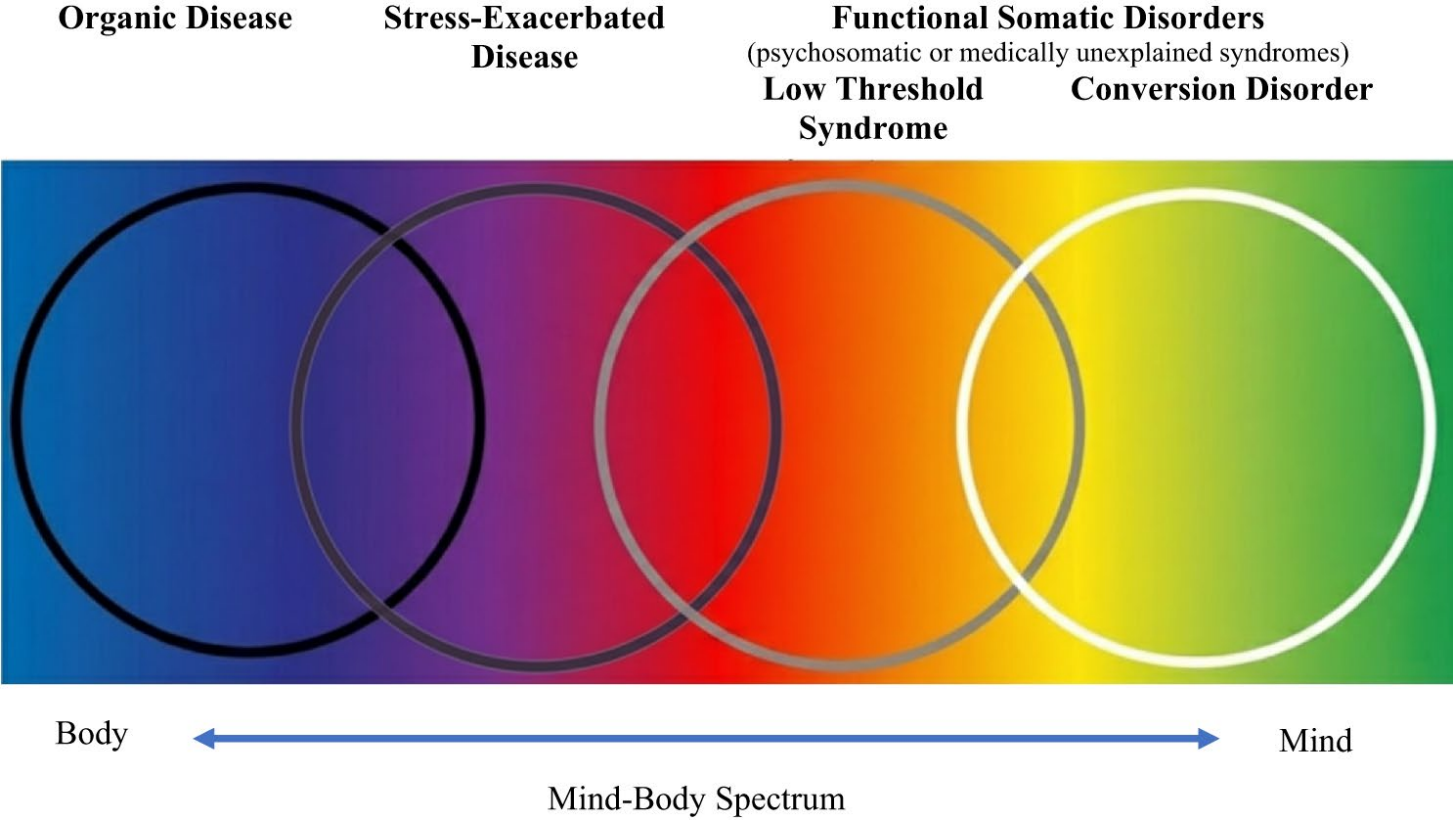
The Mind-Body Spectrum: A Conceptual Framework for Understanding Physical and Psychological Symptom Relationships

The DCGs in this study were developed in relation to a theoretical model for categorizing physical symptoms based on their underlying psycho-physiological mechanisms. This model has been discussed in a previous article, entitled “The Four-Cluster Spectrum of Mind-Body Interrelationships” [25]. Table 1 presents the four clusters and their definitions, alongside alternative terms used in the literature and clinical examples for each cluster. Figure 1 presents the four clusters along the mind-body spectrum of conditions, illustrating overlaps among clusters and positioning FSD as a term encompassing both low-threshold syndrome (cluster 3) and conversion disorder (cluster 4).

**Table 1:** Classification of four clusters along the mind-body spectrum: definitions, alternative terms, and clinical examples

Cluster	Definition	Alternative terms	Clinical example
**1. Organic Disease **	Any disease in which there is a physical change in the structure of an organ or part [26]	Structural Disease	Cerebrovascular disease
**2. Stress-Exacerbated Diseases**	A disease precipitated by activation of the stress system and chronic inflammation [27]	Inflammatory Disease	Migraine
**3. Low Threshold Syndrome**	Somatic symptoms that arise from changes in the central nervous system that alter its processing of sensory stimuli, resulting in hyperalgesia, allodynia, and sensory-motor hyperresponsiveness [28]	Central Sensitization Syndrome, Functional Somatic Disorder (FSD), Medically Unexplained Symptoms (MUS), Psychosomatic Disorder and Functional Neurologic Disorder (FND)	Fibromyalgia
**4. Conversion Disorders**	A mental disorder characterized by the conversion of mental conflicts into somatic symptoms [29]		Psychogenic Non-Epileptic Seizures (PNES)

Based on the four-cluster model, the following five diagnosis classification groups (DCGs) were hypothesized to demonstrate markedly different decreases in HUC after multidisciplinary treatment:

1. * Organic Disease (OD)* – Patients *without* FSD, treated at the clinic for organic neurologic conditions (e.g., cerebral vascular accident).
2. * Stress-Exacerbated Diseases (SED)* – Patients treated at the clinic for organic disorders exacerbated by stress (e.g., migraine).
3. * Functional Somatic Disorder with Organic Background (O/FSD)* – Patients treated primarily for FSD but with an additional organic diagnosis (i.e., cancer, renal failure, congestive heart failure, etc.).
4. * Simple Functional Somatic Disorder (S/FSD)* – Patients with an FSD diagnosis devoid of complicating factors.
5. * Difficult Functional Somatic Disorder (D/FSD)* – Patients treated with either (a) a severe FSD diagnosis (e.g., complex regional pain syndrome) or (b) high levels of emotional distress (e.g., depression, anxiety).

Of note, the first two DCG (OD and SED) map precisely onto the four-cluster model. The following three do not. This is because despite different psychological mechanisms implicated in low threshold syndromes and conversion disorders, it remains difficult to establish a method of differential diagnosis for these clusters via medical investigation, so that the current study did not attempt to distinguish between them. Additionally, the purpose of creating DCG in this study was to determine whether different groups of FSD patients could be easily identified via EMR whose HUC would be affected differently by multidisciplinary treatment. A more nuanced distinction between groups of patients treated for FSD was established with this consideration in mind.

After dividing the study population into DCG, demographic data and HUC were compared for the year prior-to and after patients’ initial FNC visit. The first visit was chosen as the beginning of the intervention, even though some of the adjunct multidisciplinary services did not take place immediately. This is because the first visit already had an immediate therapeutic effect, as this was when the neurologist explained the psycho-physiological nature of patients’ symptoms, providing reassurance and taking time for patient-education and questions. Patients were referred to additional therapy as needed, occurring over the following months. All analyses were performed for the total population as well as for individual DCG.

The study was approved by the Institutional Review Boards of both CHS and Soroka Medical Center. Informed consent was not required, as this was a retrospective study using anonymized data.

## Healthcare utilization and costs

In Israel, all citizens and permanent residents are entitled to receive a National List of Health Services (NLHS). Healthcare utilization and costs were evaluated from the perspective of the healthcare provider and only direct costs were measured. Costs are presented in Israeli Shekels (ILS); exchange rate 1 USD = 3.5 ILS. Encounters were defined as billable contacts between patients and health professionals whose services were covered by CHS. Encounters were computerized and time-stamped, enabling utilization to be looked at over time and stratified by encounter type and by site of care delivery: (a) specialist consultations, (b) hospital care (for visits priced by number of days hospitalized), (c) diagnostic tests (CT scans, MRI, ultrasound, etc.), (d) prescribed medications, (e) laboratory tests, (f) surgical procedures by procedure related group, (g) services provided by other healthcare disciplines (physical therapy, social work etc.), (h) medical equipment and (j) total costs per patient. Additional information collected included medical diagnoses or underlying conditions that influenced healthcare utilization. The cost of FNC treatment was covered by CHS under the NLHS and the Israeli mental-health reform (July 1 st, 2015), through which insurance responsibility for mental health services was transferred to the HMOs. Utilization and costs related to treatment at the FNC were analyzed both separately and as part of the patients’ overall medical care.

### Data analysis

As this was a retrospective study in which patients interacted with the medical system at different times, the index date was the date of the first visit to the FNC, coinciding with the beginning of multidisciplinary treatment. Changes in utilization and costs were calculated as the ‘year after FNC treatment began’ minus the ‘year before FNC treatment began’. Hence, a negative cost or utilization unit indicates a decrease over the course of treatment. Total healthcare costs, as well as specific utilization and cost categories were presented as mean and standard deviation (SD) or median and interquartile range (IQR). Due to the non-normal distribution of cost variables, non-parametric Wilcoxon signed rank tests were used to compare pre- and post FNC treatment This was done for the total population and separately for each DCG.

To identify patients with post-treatment cost reductions, a binary variable was defined identifying those whose post-treatment costs were lower than their pre-treatment costs. The percentage of “reduced-cost” patients is presented along with a comparison of the different DCG using a Chi-square test. Additionally, the study population was stratified by their total pre-treatment median cost into high or low baseline cost groups. A Wald test was used to compare the rate of reduced-cost patients between high and low baseline groups. Lastly, a multivariable logistic regression was performed to identify factors related to reduced-cost patients. The analysis tested the association between diagnostic classification groups (DCG) and the probability of reduced-cost patients. Therefore, the dependent variable was defined as all patients experiencing a decrease in annual total cost pre/post treatment. The model also adjusted for age, gender, socioeconomic status and baseline-costs. As a sensitivity analysis, this multivariable regression was repeated for patients with significantly reduced costs (those with a 5% or more decrease from original costs).

P-values < 0.05 indicated statistical significance in all analyses. Data analyses were performed using SAS 9.4 (SAS Institute Inc., Cary, NC, USA).

## Results

### Demographic characteristics

Among the 924 patients treated at the FNC between 2011 and 2016, 685 were insured by CHS and their data were analyzed. Baseline characteristics of the study population (Table 2) show that most were female and married. Detailed descriptive statistics including measures of variability for all demographic and clinical characteristics can be found in Supplementary Table S1. The study population was about evenly distributed between DCGs. There were several demographic differences between groups, mostly centered around age and sex. The SED group had the highest percentage of female patients. The OD group had relatively fewer females and was significantly older in age (Table 2). The S/FSD group was the youngest in age. Socioeconomic status was similar across DCG. Additional differences between groups were seen regarding underlying medical conditions and health behaviors. In the OD, D/FSD, and O/FSD groups, 30% of patients smoked, while in the S/FSD and SED groups, only 20% smoked. In both groups with people with organic diseases (OD and O/FSD), around 20% of patients had diabetes, while for the non-organic groups (D/FSD, S/FSD and SED), the percentage of people with diabetes was 3%–8%. Similar trends were seen regarding other common conditions, such as hypothyroidism, hyperlipidemia and hypertension.

Although these demographic differences were apparent on univariate analysis, they did not remain significant on multivariate analysis (Fig. 3) nor did they significantly affect patients’ responses to treatment.

**Table 2:** Key demographic characteristics and medical background for patients treated at the Functional Neurologic Clinic (FNC)

*N*	Total population	OD	D/FSD	O/FSD	S/FSD	SED
	685	207	110	125	143	100
Mean age (years)	46.1	52.5	43.5	48.8	38.7	42.2
Female (%)	69.3	60.0	72.5	67.2	71.0	85.6
Married (%)	57.4	54.1	57.8	62.4	50.0	68.0
Current smoker (%)	30.2	35.7	30.0	36.0	23.8	21.0
**Common medical diagnoses (%)**						
Hyperlipidemia	45.8	60.9	30.9	60.8	26.6	40.0
Arthropathy	44.8	52.2	40.9	62.4	32.2	30.0
Hypertension	24.5	42.5	13.6	32.8	9.8	10.0
Diabetes	13.9	24.2	8.2	22.4	3.5	3.0
Hypothyroidism	11.4	14.5	8.2	16.8	8.4	6.0

OD = Organic Disease; D/FSD = Difficult Functional Somatic Disorder; O/FSD = Functional Somatic Disorder with Organic Disease; S/FSD = Simple Functional Somatic Disorder; SED = Stress Exacerbated Disease

## Characterization of healthcare utilization and costs during FNC treatment

Patterns of HUC were consistent with the multidisciplinary approach used at the FNC (Table 3). Most FNC visits were neurological consultations, with an annual average of 2.33 visits per patient, with a skewed distribution (Supplementary Table S2-3). Multidisciplinary services included psychological and physical therapy, hydrotherapy, participation in MBCT groups, and social work consultations. Costs reflected utilization rates, with neurological consultations having the highest mean annual cost followed by psychological therapy and physiotherapy. There were no significant differences in FNC utilization among DCG, except that the D/FSD group had higher utilization and costs rates for mental health services, when compared with the other DCG.

**Table 3:** Annual FNC service utilization and costs per patient by diagnostic group

Service type	OD	D/FSD	O/FSD	S/FSD	SED	Total	Mean cost
Neurologist	2.3	2.2	2.4	2.3	2.4	2.3	268
Physical therapy	0.9	1.1	0.9	1.4	0.6	1.0	107
Psychologist	0.2	0.5	0.4	0.2	0.2	0.3	264
MBCT group	0.5	1.1	0.6	0.6	0.6	0.6	41
Hydrotherapist	0.3	0.4	0.1	0.3	0.0	0.2	12
Social worker	0.0	0.01	0.02	0.01	0.0	0.01	1

Costs noted in Israeli Shekel (ILS). OD = Organic Disease; D/FSD = Difficult Functional Somatic Disorder; O/FSD = Functional Somatic Disorder with Organic Disease; S/FSD = Simple Functional Somatic Disorder; SED = Stress Exacerbated Disease

## Changes in healthcare utilization and cost after FNC treatment

HUC were measured during the years before and after beginning FNC treatment. Utilization and costs decreased over the course of treatment in several areas. The rate of diagnostic tests such as MRI or CT scans decreased for the entire study population by an average of 4.4 tests per year, consistent across all DCG. Other changes in utilization can be seen across various categories (Table 4). Total annual costs decreased for the study population by a mean of ILS − 1,367 (*P*<0.0001). Regarding individual DCG, cost reductions were significant for the O/FSD, S/FSD and SED groups. While in specific services (i.e., hospitalizations and diagnostic tests) costs were reduced for the D/FSD group, there were no significant changes in total costs for this group, nor for the OD group (Supplementary Table S4-5).

The categories with the most significant cost reductions in the entire FNC population and across DCGs were diagnostic procedures, laboratory tests and hospitalizations. The cost categories with the smallest decreases were specialist consultations and services provided by other health professionals. This is likely because visits to the neurologist and other health professionals (e.g., physical therapist, social worker) were included in these categories, and so costs were more likely to increase.

**Table 4:** Changes in healthcare utilization and costs after FNC treatment

Category	OD	D/FSD	O/FSD	S/FSD	SED	Total
Total costs	267	−2,129	−4,074†	−1,096†	−912*	−1,367‡
Hospitalizations	183‡	−2,552*	−4,923‡	−1,765‡	−694*	−1,723‡
Specialist consultations	1,132‡	587‡	1,016†	310‡	242‡	722‡
Diagnostic tests	−234*	−497*	−1,013‡	−473†	−419*	−495‡
Surgical procedures	−173	278	461	742	−172	217
Medical equipment	−252	0	683	−0.1	−0.7	48
Services by health professionals	51	100	−92	98*	23	38*
Laboratory tests	−93*	−68*	−27	−28	2	−49‡
Medications	−200‡	14	−5	257	172	19‡
Other	−147†	−57*	−174	−235*	−65	−144‡

Values are mean changes in Israeli Shekels (ILS)

Negative values indicate reduction. *P < 0.05, †*P* < 0.01, ‡*P* < 0.001

### Identifying patients with reduced costs

**Fig. 2:**
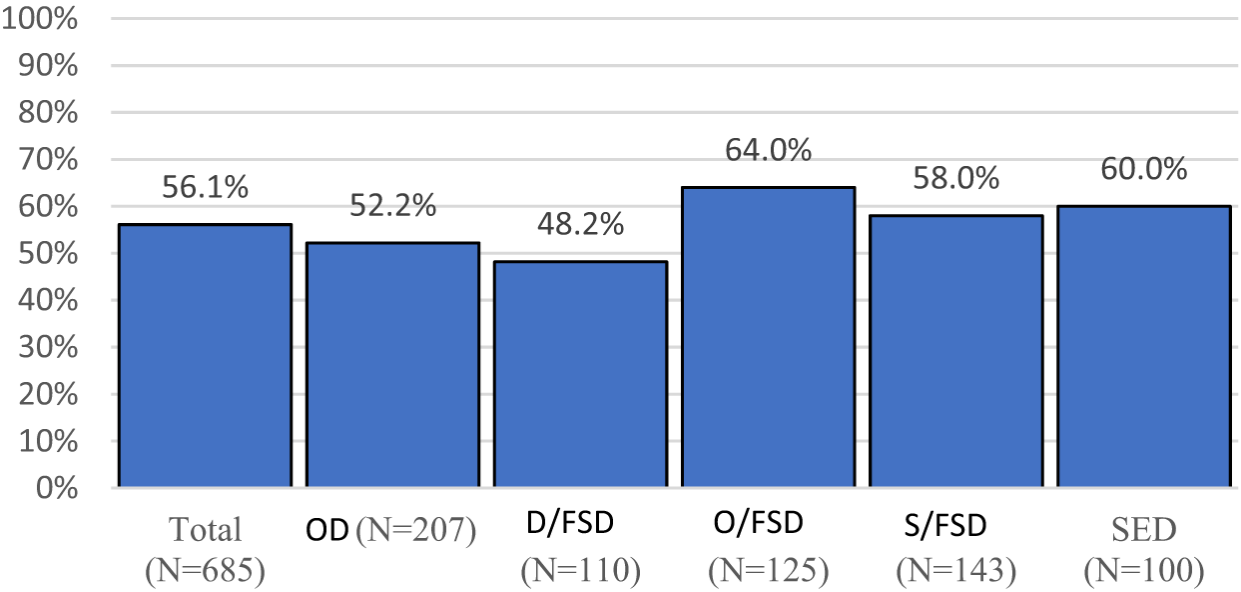
Percentage of patients with cost reductions by Diagnosis Classification Group (DCG). OD = Organic Disease; D/FSD = Difficult Functional Somatic Disorder; O/FSD = Functional Somatic Disorder with Organic Disease; S/FSD = Simple Functional Somatic Disorder; SED = Stress Exacerbated Disease

In order to identify which patients exhibited cost reductions after FNC treatment, patients were defined as “reduced” or “not reduced” based on whether their costs increased or decreased after treatment (Fig. 2). Over the study period, costs decreased in over half of the patients in all DCG, except D/FSD (48.2%). A reduction in costs was seen for 56.1% of the total population.

Due to the high variability of baseline costs across DCG and their influence on post-treatment cost reductions, this analysis was repeated after stratifying the study population by high/low baseline costs. High-cost patients were defined as those with baseline costs above the median annual cost (ILS 5,854), whereas low-cost patients were those whose baseline costs were under the median. High-cost patients had a significantly higher rate of cost reduction when compared to low-cost patients (72.2% vs. 39.9%). This held true for the population as a whole and for each DCG (*P*<0.0001 for all comparisons using Wald tests).

Additionally, when stratifying the population by baseline costs, significant differences could be seen between DCG. For high-cost patients, cost reduction rates were significantly higher for the O/FSD (81%), S/FSD (77.6%) and SED (80%) groups, and lower for the OD (64.5%) and D/FSD (69.8%) groups. For low-cost patients, this pattern remained consistent for the S/FSD (47.9%) and SED (53.3%) groups when compared to the OD (25.8%) and D/FSD (34.3%) groups. However, the low-cost patients from the O/FSD (29.3%) group had lower reduction rates, similar to the OD and D/FSD groups.

**Fig. 3:**
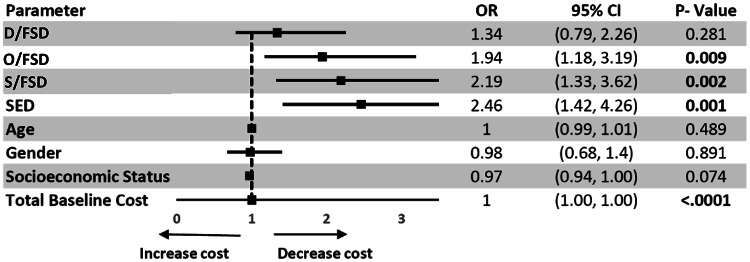
Association between DCG and the probability of reduced-cost patients following multidisciplinary treatment. Multivariable logistic regression showing odds ratios (with 95% CI) for reduced healthcare costs after treatment, adjusted for demographics and baseline costs. The Organic disease (OD) group serves as the reference. D/FSD = Difficult Functional Somatic Disorder; O/FSD = Functional Somatic Disorder with Organic Disease; S/FSD = Simple Functional Somatic Disorder; SED = Stress Exacerbated Disease

A multivariable, logistic regression was performed to ascertain the effects of age, sex, socioeconomic status and each of the DCG on costs (Fig. 3). Compared to the OD group, costs were reduced for a significantly higher percentage of patients in the SED, S/FSD and O/FSD groups. Results were not significant for the D/FSD group and were not affected by age, gender and socioeconomic status. Since grouping patients based solely on whether or not costs were reduced could be problematic (e.g., if costs increased or decreased by only ILS 1, there would be no real difference between patients), the multivariable analysis was repeated for patients with significantly reduced costs (defined as patients with a decrease of at least 5% of original costs) and yielded similar results.

## Discussion

### Summary of main results

This study aimed to identify changes in healthcare utilization and costs (HUC) among patients with Functional Somatic Disorders (FSD) receiving multidisciplinary treatment that integrates medical and psychological care. In attempting to analyze which patients would most benefit from this approach, the study population was divided into diagnosis classification groups (DCGs). Decreases in HUC were observed in the overall population and to varying degrees in the different DCGs, indicating that baseline diagnostic variables may be helpful in determining where reductions are likely to occur. Overall, cost-reductions were most consistent regarding diagnostic tests, laboratory tests and hospitalizations.

Total costs decreased among all DCGs, except for people with organic diseases (OD) and with difficult FSD (D/FSD). These findings are consistent with the four-cluster model, as MBTs are not the treatment of choice for patients with OD, and reductions in healthcare utilization and costs were not expected for this group. These findings are similarly unsurprising regarding the D/FSD group. Patients with D/FSD received more intensive multidisciplinary treatment at the FNC and incurred more intensive treatment costs. This likely made it harder to demonstrate cost reductions. In this regard, data from the FNC showed that indeed the only significant difference in FNC utilization and cost among DCG, was that the D/FSD group had higher HUC rates for mental health services, when compared with other DCG (see Supplementary Table S3). The percentage of patients with cost-reductions for HUC was consistently highest among the simple FSD (S/FSD) and stress exacerbated disorders (SED) groups, and lowest for the OD and D/FSD groups. These findings are particularly interesting in regard to the SED group, as these patients are often treated solely through bio-medical interventions. The current study demonstrates that incorporating MBTs into the treatment of SEDs (e.g., migraines, autoimmune diseases etc.) is likely advantageous and cost-effective. Patients in the O/FSD group with high baseline costs achieved substantial cost reductions, whereas patients with low baseline costs showed only limited reductions. A similar pattern was seen in the logistic regression, demonstrating high reduction-rates for patients in the SED, S/FSD and O/FSD groups when compared to the OD group. Reduction-rates were not significantly different between the OD and D/FSD groups, nor were they influenced by demographics.

### Comparison with existing literature

Multidisciplinary treatment models have shown positive results for a variety of somatic symptoms associated with FSD, such as generalized chronic pain, [6, 31] lower back pain [31, 32] and neck pain [29, 33]. A multidisciplinary stepped-care clinic for Non-Cardiac Chest Pain integrating cardiological and psychological care, led to improvements in illness perceptions, depression, anxiety, somatic symptoms, and exercise avoidance [34]. Such clinical benefits have been shown to parallel decreases in HUC [35].

In this study, HUC reduction rates for the total population were approximately 50%. A similar retrospective pre- and post-intervention database analysis of relaxation and resilience training for a diverse cohort of patients found comparable reductions in mean healthcare utilization rates (43%).^24^ However, their study was not focused on people with FSD or similar disorders. A systematic review by Hartman et al. [36] found that clinical improvement rates varied widely from 30 to 75% for patients with medically unexplained symptoms (MUS) or hypochondriasis. However, their review focused on symptom improvement rather than healthcare utilization and emphasized a lack of empirical evidence regarding prognostic factors.

The current study found the most consistent decreases centered around diagnostic processes (e.g., MRI, CT, laboratory tests, etc.) and hospitalizations. Although economic reviews have found that multidisciplinary teams are cost-effective, little information has been available regarding in-depth analyses of cost-categories [37, 38]. A cost minimization analysis of patients with functional disorders undergoing CBT, similarly found that the intervention group had consistently lower healthcare costs, with significant reductions regarding in-patient hospital care in the year post-treatment [21]. An economic review of MUS concluded that across cost-of-illness studies, a high portion of direct costs stemmed from inpatient treatments (e.g., hospitalizations, surgeries) and diagnostic procedures [37]. The current findings are in accord with those trends, indicating that multidisciplinary care for people with FSD may reduce HUC by reducing unnecessary diagnostics and hospitalizations.

This study was unique in dividing FSD into prognostic groups based on diagnostic classification. Few other attempts have been made to create specific prognostic systems for FSD. One of the most comprehensive efforts was made by Danish researchers who divided Bodily Distress Syndrome (parallel to FSD) into groups based on symptom-severity, entitled the “Functional Somatic Disorder (FSD) classification”. [1, 39, 42] Three categories emerged from the FSD assessing how likely symptoms are to persist, impacting patients’ quality of life and HUC [43] (1) Multi-system FSD (also termed “symptom disorder”): patients experiencing multiple persistent symptoms across different physiological systems causing disability, extensive HUC, comorbid psychiatric distress and a poor prognosis. (2) Single-system FSD (also “recurrent symptoms”): A persistent cluster of symptoms with a moderate prognosis, occurring in a single bodily system and often related to existing diagnostic labels (e.g., fibromyalgia, IBS). These symptoms may include a combination of unexplained symptoms and symptom-congruent medical conditions (e.g., IBS and colitis). (3) Single-symptom FSD (also “self-limiting symptoms”): isolated symptoms (e.g., headache, dizziness, tinnitus), often with a good prognosis. This classification is purported to be clinically beneficial, despite the authors’ acknowledgment that it has yet to be empirically verified [1, 43].

The current study may offer initial corroboration for the Danish FSD classification. Both models looked at diagnosis-based classification. Since many of the same variables were considered, a comparison could be drawn between our DCG classifications and the Danish FSD classification. In comparing systems, the D/FSD group would parallel Multi-system FSD, representing challenging cases of FSD, less likely to have a good prognosis. Some similarities can be drawn between the O/FSD group and Single-system FSD, given that both groups have an additional medical condition. Finally, the S/FSD group would parallel Single-symptom FSD, where a good prognosis is more likely to occur. Understandably, the OD and SED groups do not exist in the Danish FSD classification, as they are particular to the four-cluster model.

Despite the similarities between these models, there are also differences. One central difference is that the Danish FSD classification defines severity by the number of symptoms/systems involved. In lieu of this, our DCG was based on the hypothesis that some types of FSD (e.g., fibromyalgia and CRPS) are more severe than others. This difference, however, may not be as significant as it first appears. This is because the more severe conditions in the DCG classification, such as fibromyalgia, have been shown to have high co-morbidity with other FSDs [44–46]. It is likely then, that there is overlap between defining severity based on the number of symptoms and the assertion that diagnoses such as fibromyalgia are more severe. Additionally, the existence of depression alongside fibromyalgia symptoms has been associated with greater severity [47]. Lastly, in our model for the O/FSD group, it was not required that the functional and organic conditions would be from the same system as was required for its parallel in the Danish classification.

In summary, this study supports the notion that baseline diagnostic criteria could help elucidate how different FSD patients are affected by multidisciplinary treatment, similar to the FSD classification. The D/FSD group, representing severe cases, had the lowest reduction-rates, similar to patients with OD. The S/FSD group had the highest reduction rates, similar to those of the SED group. This similarity may represent the effectiveness of multidisciplinary treatment in reducing HUC for headache patients, as tension-type headaches were common among the S/FSD group and migraines in the SED group. The O/FSD group’s reduction-rates varied based on baseline costs. This is likely statistical rather than clinical, as larger cost reductions are expected for high-cost patients. Nonetheless, it demonstrates that the O/FSD group constitutes a middle-ground between severe and mild FSD.

### International context and implementation challenges

The Israeli experience with multidisciplinary FSD care reflects broader international trends and challenges documented across European healthcare systems. Many pain treatment centers around the world still utilize a bio-medical model when it comes to treating chronic pain and FSD, primarily offering such treatments as nerve-blocks and injections. Bio-medical treatments are often the go-to in many pain treatment centers, despite some having been shown to be no more effective than placebos [48, 49]. There are likely many reasons for this. Some may be theoretical and historical, such as the use of an anachronistic bio-medical paradigm to guide treatment decisions. However, other considerations are likely financial and systemic. Bio-medical treatments are often more lucratively reimbursed, do not necessitate additional staff or training, and do not require physicians to invest time and effort in patient education [11].

This study demonstrates the importance and cost-effectiveness of incorporating MBTs into routine medical practice, implementing such changes is not simple. Research from the EURONET-SOMA network identifies persistent challenges, including: defining clinically useful diagnostic terms, implementing guideline recommendations into routine care, and developing effective dissemination strategies [50]. Implementation obstacles identified in Germany, Italy, the Netherlands, and Poland include inadequate training, stigmatization, financial constraints, and understaffing, which critically affect multidisciplinary integration [51]. Healthcare professionals across these countries consistently advocate for better integration of psychological care while noting knowledge gaps often rooted in traditional bio-medical models.[52] Similar to Israel’s context, international studies indicate that primary and secondary care collaboration faces systemic barriers, including unclear roles, time pressures, lack of unified protocols, and insufficient workforce capacity [53]. However, successful models demonstrate that healthcare providers (including internal medicine specialists) with proper training and sufficient time can effectively diagnose FSD [54]. These international experiences validate the relevance of our DCG framework for healthcare systems worldwide seeking evidence-informed approaches to optimize FSD care delivery.

### Health policy implications

This research demonstrates that multidisciplinary care resulted in cost reductions for more than half of participating patients with functional somatic disorders (FSD), with response patterns that were predictable based on diagnostic classification. These novel findings offer healthcare systems in Israel and internationally a framework for optimizing resource allocation. Our results support four key policy recommendations for implementing cost-effective, personalized care pathways. First, healthcare systems should allocate resources toward establishing multidisciplinary treatment centers. In Israel, only a handful of specialized services exist nationwide, and most physicians treating FSD face systemic obstacles, including a lack of competent professionals to refer to, due to insufficient resource allocation toward specialized treatment centers [14, 17]. Our results provide novel evidence for the financial viability of such investments by demonstrating greater cost reductions, specifically among patients with Simple FSD and Stress-Exacerbated Disease.

Second, healthcare systems should invest in training physicians, psychologists, and allied health professionals in multidisciplinary, mind-body approaches to treating FSD. While Israel’s Ministry of Health resolution on Standards for Medical Psychological Treatment [55] was a step in the right direction, evidence from a European study conducted across Denmark, the Netherlands, Norway, and the UK demonstrates that positive engagement with patients with functional somatic symptoms relies on healthcare professionals having a shared understanding of symptom mechanisms [56]. Our findings support this by showing that FSD patient subgroups respond variably to multidisciplinary care, highlighting the need for trained professionals who can tailor approaches to specific presentations. Alongside physicians interested in functional medicine, medical psychologists, who are already trained to work in healthcare settings and to use mind-body techniques, should receive specific education in applying their therapeutic acumen to work with FSD. They could potentially be involved in various levels of care; both staffing tertiary treatment centers like the FNC, as well as engaging in training for physicians in primary and secondary care, as yet unfamiliar with multidisciplinary treatment for FSD.

Third, healthcare professionals treating FSD should use information in electronic medical records to assist in early identification and classification of patients (e.g., baseline diagnostic criteria, comorbid conditions, and symptom severity patterns). The Danish FSD classification system demonstrates that prognosis-based recognition using electronic medical records enables systematic categorization of patients into severity groups that predict treatment outcomes [43]. Our DCG framework builds on this approach by providing a validated system for patient selection and care routing based on readily available diagnostic information in electronic health records.

Lastly, treatment for patients with FSD should be tailored to individual needs, with personalized approaches and programs for distinct patient groups. Evidence from integrated care models in the UK demonstrates that multidisciplinary specialty clinics facilitate cost-effective care while minimizing fragmentation through personalized treatment pathways [57]. Our findings demonstrate that Simple FSD and SED patients achieve higher cost reduction outcomes compared to Difficult FSD patients, providing evidence for developing stratified care approaches based on diagnostic classification.

### Strengths and limitations

This study has several strengths and limitations. Strengths included the use of high-quality EMR data. Data were obtained from the insurance provider, thereby avoiding recall and response bias associated with self-report surveys. Patients presented with a range of FSDs, increasing the clinical relevance of the findings. Another strength was the correction for the non-normal distribution of the study population. A recent meta-analysis of psychologically based interventions for FSD noted that many of the studies reviewed exhibited non-normal distributions and skewness, with no evidence of attempts to correct for these [22]. Because the cost variables in this study were not normally distributed, comparisons were performed using nonparametric Wilcoxon tests. Additionally, although baseline costs influenced the results, it is more difficult to demonstrate cost reductions among patients with low initial costs; therefore, this was controlled for using logistic regression. In addition, patients were stratified into high- and low-baseline-cost groups, demonstrating that although high-baseline-cost patients had greater absolute reductions, low-baseline-cost patients had similar percentage reductions.

This study also had important limitations. There was no control group, and so this study cannot provide information on the effectiveness of multidisciplinary treatment compared to usual care. An important related limitation is the absence of a control group receiving neurological consultation alone without multidisciplinary services, which would help isolate the specific contribution of multidisciplinary interventions versus the therapeutic effect of diagnostic clarification and reassurance provided during the initial consultation. A recent randomized controlled trial (RCT) of forty patients demonstrated the effectiveness of multidisciplinary treatment in functional disorders [58]. Our findings in a larger cohort (*N* = 685) align with their results while providing essential evidence to policymakers on HUC. This study did not capture multidisciplinary treatments patients may have received through private insurance or out-of-pocket payments prior to FNC treatment, which could have influenced baseline healthcare utilization patterns and treatment responsiveness. Additionally, this was a retrospective study, and so results may be less reliable than if they had been generated through a prospective study design. Although the OD group could be viewed, to some extent, as a comparison group, it was not preselected or matched on demographic variables. We attempted to correct for this by performing a multivariable regression, demonstrating that DCG were influential beyond demographics. Additionally, this study examined HUC alone and did not include clinical measures. Finally, this was a single-center study; therefore, the results may not be generalizable to other teams or facilities that provide different forms of multidisciplinary treatment.

## Conclusions

The results of this study indicate that multidisciplinary treatment combining medical and psychological care can potentially benefit patients with FSD. Although the primary goal of a multidisciplinary approach is to improve quality of life for patients with FSD, adopting a multidisciplinary team model can also yield financial benefits for the healthcare system. The information presented here can be used to design an algorithm that empowers treatment teams and policymakers to select patients most likely to benefit from a multidisciplinary approach. Future prospective studies are needed to support these findings.

## Supplementary Information

Supplementary Material 1.

## Data Availability

The datasets used during the current study are not publicly available due to privacy restrictions from Clalit Health Services but are available from the corresponding author upon reasonable request and with permission from Clalit Health Services.
