# Supplementary material for: Who benefits from multidisciplinary care in functional somatic disorders? Identifying cost-effective patient selection through diagnostic classification groups
Source: Isr J Health Policy Res. 2026 Mar 18;15:9. doi: 10.1186/s13584-026-00750-7 (PMC12997945; doi:10.1186/s13584-026-00750-7)
Supplement: Supplementary file 1 — Supplementary Material 1. [file 13584_2026_750_MOESM1_ESM.docx]

**Supplementary Materials for Healthcare Utilization and Costs for Functional Disorders**

**Supplementary Table S1. Complete demographic and clinical characteristics by diagnostic group**

|  | **Total Population** | | **OD** | | **D/FSD** | | **O/FSD** | | **S/FSD** | | **SED** | |
| --- | --- | --- | --- | --- | --- | --- | --- | --- | --- | --- | --- | --- |
| **N (%)** | 685 (100%) | | 207 (30.2%) | | 110 (16.1%) | | 125 (18.3%) | | 143 (20.9%) | | 100 (14.6%) | |
|  | **Mean (SD)** | **Median (IQR)** | **Mean (SD)** | **Median (IQR)** | **Mean (SD)** | **Median (IQR)** | **Mean (SD)** | **Median (IQR)** | **Mean (SD)** | **Median (IQR)** | **Mean (SD)** | **Median (IQR)** |
| **Age** | 46.07 (16.58) | 47.42 (33.08, 57.99) | 52.47 (17.74) | 54.56 (38.61, 64.76) | 43.46 (14) | 42.48 (33.35, 52.96) | 48.79 (16.1) | 50.57 (38.11, 59.99) | 38.72 (15.01) | 35.14 (26.72, 52.4) | 42.23 (13.4) | 42.65 (33.08, 52.5) |
| **Socioeconomic Grade** | 8.18 (5.22) | 9 (4, 12) | 8.16 (5.3) | 9 (6, 12) | 7.67 (5.35) | 8 (3, 12) | 7.9 (4.59) | 8 (6, 11) | 8.57 (4.88) | 9 (6, 12) | 8.59 (6.13) | 10 (0, 13) |
|  | **N** | **%** | **N** | **%** | **N** | **%** | **N** | **%** | **N** | **%** | **N** | **%** |
| **Male** | 205 | 30.7% | 82 | 40.0% | 28 | 27.5% | 41 | 32.8% | 40 | 29.0% | 14 | 14.4% |
| **Female** | 462 | 69.3% | 123 | 60.0% | 74 | 72.5% | 84 | 67.2% | 98 | 71.0% | 83 | 85.6% |
| **Not Married** | 284 | 42.6% | 94 | 45.9% | 43 | 42.2% | 47 | 37.6% | 69 | 50.0% | 31 | 32.0% |
| **Married** | 383 | 57.4% | 111 | 54.1% | 59 | 57.8% | 78 | 62.4% | 69 | 50.0% | 66 | 68.0% |
| **Non-smoker** | 436 | 63.6% | 116 | 56.0% | 70 | 63.6% | 72 | 57.6% | 105 | 73.4% | 73 | 73.0% |
| **Current smoker** | 207 | 30.2% | 74 | 35.7% | 33 | 30.0% | 45 | 36.0% | 34 | 23.8% | 21 | 21.0% |
| **Former smoker** | 42 | 6.1% | 17 | 8.2% | 7 | 6.4% | 8 | 6.4% | 4 | 2.8% | 6 | 6.0% |
| **Common Medical Diagnoses** | | | | | | | | | | | | |
| **Hyperlipidemia** | 314 | 45.8% | 126 | 60.9% | 34 | 30.9% | 76 | 60.8% | 38 | 26.6% | 40 | 40.0% |
| **Arthropathy** | 307 | 44.8% | 108 | 52.2% | 45 | 40.9% | 78 | 62.4% | 46 | 32.2% | 30 | 30.0% |
| **Obesity** | 190 | 27.7% | 73 | 35.3% | 34 | 30.9% | 40 | 32.0% | 30 | 21.0% | 13 | 13.0% |
| **Hypertension** | 168 | 24.5% | 88 | 42.5% | 15 | 13.6% | 41 | 32.8% | 14 | 9.8% | 10 | 10.0% |
| **Reflux Esophagitis, Gastritis, Duodenitis** | 140 | 20.4% | 51 | 24.6% | 24 | 21.8% | 33 | 26.4% | 17 | 11.9% | 15 | 15.0% |
| **Diabetes** | 95 | 13.9% | 50 | 24.2% | 9 | 8.2% | 28 | 22.4% | 5 | 3.5% | 3 | 3.0% |
| **Other kidney disease** | 79 | 11.5% | 28 | 13.5% | 9 | 8.2% | 24 | 19.2% | 8 | 5.6% | 10 | 10.0% |
| **Hypothyroidism** | 78 | 11.4% | 30 | 14.5% | 9 | 8.2% | 21 | 16.8% | 12 | 8.4% | 6 | 6.0% |
| **Asthma** | 69 | 10.1% | 25 | 12.1% | 7 | 6.4% | 13 | 10.4% | 15 | 10.5% | 9 | 9.0% |
| **Osteoporosis** | 68 | 9.9% | 28 | 13.5% | 11 | 10.0% | 18 | 14.4% | 8 | 5.6% | 3 | 3.0% |
| **Mortality** | 10 | 1.5% | 8 | 3.9% | 0 | 0.0% | 1 | 0.8% | 0 | 0.0% | 1 | 1.0% |

Note: OD = Organic Disease; D/FSD = Difficult Functional Somatic Disorder; O/FSD = Functional Somatic Disorder with Organic Disease; S/FSD = Simple Functional Somatic Disorder; SED = Stress Exacerbated Disease.

**Supplementary Table S2. Functional neurologic clinic utilization by diagnosis classification group (DCG)**

Mean values with standard deviation in brackets

| **Variable** | **Total (N=685)** | **OD (N=207)** | **D/FSD (N=110)** | **O/FSD (N=125)** | **S/FSD (N=143)** | **SED (N=100)** |
| --- | --- | --- | --- | --- | --- | --- |
| **Total visits** | 2.77 (2.41) | 2.95 (2.37) | 2.61 (2.21) | 2.95 (2.86) | 2.55 (2.12) | 2.67 (2.46) |
| **Neurologist** | 2.33 (1.52) | 2.31 (1.32) | 2.23 (1.44) | 2.41 (1.71) | 2.34 (1.61) | 2.38 (1.62) |
| **Physical Therapist** | 0.97 (2.49) | 0.89 (2.41) | 1.05 (2.51) | 0.86 (2.06) | 1.38 (3.28) | 0.62 (1.63) |
| **Social Worker** | 0.01 (0.09) | 0  (0.07) | 0.01  (0.1) | 0.02 (0.15) | 0.01 (0.08) | 0  (0) |
| **Psychologist** | 0.26 (0.93) | 0.21 (0.79) | 0.47 (1.17) | 0.38 (1.21) | 0.17 (0.73) | 0.15 (0.67) |
| **Hydro-therapist** | 0.22  (1.7) | 0.29 (1.71) | 0.36 (2.27) | 0.1  (0.58) | 0.25 (2.31) | 0  (0) |
| **MBCT Group** | 0.62  (2) | 0.47 (1.86) | 1.08 (2.51) | 0.58 (1.77) | 0.57 (1.96) | 0.56 (1.94) |

Note: OD = Organic Disease; D/FSD = Difficult Functional Somatic Disorder; O/FSD = Functional Somatic Disorder with Organic Disease; S/FSD = Simple Functional Somatic Disorder; SED = Stress Exacerbated Disease. MBCT = mindfulness-based cognitive therapy.

**Supplementary Table S3. Mean annual FNC costs in Israel Shekel by DCG**

Mean values with standard deviation in brackets

| **Variable** | **Total (N=685)** | **OD (N=207)** | **D/FSD (N=110)** | **O/FSD (N=125)** | **S/FSD (N=143)** | **SED (N=100)** |
| --- | --- | --- | --- | --- | --- | --- |
| **Total visits** | 639.14 (1003.6) | 582.44 (877.19) | 825.23 (1172.16) | 750.83 (1301.33) | 582.94 (861.34) | 492.6 (756.75) |
| **Neurologist** | 268.05 (174.66) | 265.56 (151.65) | 256.38 (165.35) | 277.3 (196.75) | 268.6 (185.6) | 273.7 (186.21) |
| **Physical Therapist** | 107.04 (266.52) | 97.82 (261.51) | 117.08 (279.42) | 94.52 (222.01) | 149.55 (336.19) | 69.98 (184.86) |
| **Social Worker** | 1.05 (11.19) | 0.58 (8.34) | 1.09 (11.44) | 2.88 (18.44) | 0.84 (10.03) | 0 (0) |
| **Psychologist** | 263.78 (908.57) | 218.48 (788.74) | 453.01 (1116.65) | 378.35 (1201.95) | 163.96 (697.7) | 148.92 (663.29) |
| **Hydro-therapist** | 11.96 (93.59) | 15.94 (94.23) | 20 (125.05) | 5.72 (31.87) | 13.85 (127.32) | 0  (0) |
| **MBCT Group** | 41.46 (122.01) | 28.99 (103.95) | 72.73 (154.98) | 44.8 (126.65) | 33.57 (111.29) | 40  (120.6) |

Note: OD = Organic Disease; D/FSD = Difficult Functional Somatic Disorder; O/FSD = Functional Somatic Disorder with Organic Disease; S/FSD = Simple Functional Somatic Disorder; SED = Stress Exacerbated Disease. MBCT = mindfulness-based cognitive therapy.

**Supplementary Table S4. Changes in total healthcare utilization for the entire study population and per DCG**

|  |  | **Delta** | |
| --- | --- | --- | --- |
|  | **Variable** | **Mean (SD)** | **Median (IQR)** |
| **Total Population (N=685)** | **Number of Hospitalizations** | -0.43 (2.57) | 0 (-1, 0) |
|  | **Hospitalization days** | -0.56 (19.16) | 0 (-1, 0) |
|  | **Number of Surgeries** | 0.01 (0.41) | 0 (0, 0) |
|  | **Services provided by health professionals** | 0.28 (8.19) | 0 (-1, 1) |
|  | **Diagnostic tests** | **-4.4 (21.51)** | -2 (-17, 7) |
|  | **Laboratory tests** | -1.84 (12.38) | -1 (-8, 4) |
|  | **Medical equipment** | 0.18 (4.47) | 0 (0, 0) |
|  | **Other** | -0.25 (1.58) | 0 (-1, 0) |
| **OD (N=207)** | **Number of Hospitalizations** | -0.41 (3.01) | 0 (-1, 0) |
|  | **Hospitalization days** | 0.76 (33.43) | 0 (-3, 0) |
|  | **Number of Surgeries** | 0.03 (0.56) | 0 (0, 0) |
|  | **Services provided by health professionals** | 0.26 (9.89) | 0 (-1, 1) |
|  | **Diagnostic tests** | **-2.56 (25.13)** | -1 (-18, 13) |
|  | **Laboratory tests** | -1.27 (12.62) | 0 (-7, 6) |
|  | **Medical equipment** | 0.57 (8.1) | 0 (0, 0) |
|  | **Other** | -0.24 (1.53) | 0 (-1, 0) |
| **D/FSD (N=110)** | **Number of Hospitalizations** | -0.26 (1.75) | 0 (0, 0) |
|  | **Hospitalization days** | -1.02 (7.28) | 0 (0, 0) |
|  | **Number of Surgeries** | 0.03 (0.25) | 0 (0, 0) |
|  | **Services provided by health professionals** | 0.52 (8.24) | 0 (-1, 2) |
|  | **Diagnostic tests** | **-6.06 (20.94)** | -2 (-17, 6) |
|  | **Laboratory tests** | -2.37 (13.34) | -1 (-8, 4) |
|  | **Medical equipment** | 0 (0) | 0 (0, 0) |
|  | **Other** | -0.3 (1.42) | 0 (-1, 0) |
| **O/FSD (N=125)** | **Number of Hospitalizations** | -1.02 (4.02) | 0 (-1, 0) |
|  | **Hospitalization days** | -2.27 (10.1) | 0 (-2, 0) |
|  | **Number of Surgeries** | -0.01 (0.41) | 0 (0, 0) |
|  | **Services provided by health professionals** | -0.44 (8.96) | 0 (-2, 2) |
|  | **Diagnostic tests** | **-6.8 (20.63)** | -7 (-20, 6) |
|  | **Laboratory tests** | -2.62 (13.32) | -1 (-9, 4) |
|  | **Medical equipment** | 0.06 (0.72) | 0 (0, 0) |
|  | **Other** | -0.26 (1.81) | 0 (-1, 1) |
| **S/FSD (N=143)** | **Number of Hospitalizations** | -0.2 (0.89) | 0 (0, 0) |
|  | **Hospitalization days** | -0.81 (3.41) | 0 (0, 0) |
|  | **Number of Surgeries** | 0.01 (0.36) | 0 (0, 0) |
|  | **Services provided by health professionals** | 0.88 (6.74) | 0 (0, 1) |
|  | **Diagnostic tests** | **-3.24 (20.33)** | -1 (-14, 7) |
|  | **Laboratory tests** | -1.34 (11.86) | -1 (-7, 4) |
|  | **Medical equipment** | 0.01 (0.32) | 0 (0, 0) |
|  | **Other** | -0.35 (1.79) | 0 (-1, 0) |
| **SED (N=100)** | **Number of Hospitalizations** | -0.21 (1.1) | 0 (0, 0) |
|  | **Hospitalization days** | -0.29 (1.48) | 0 (0, 0) |
|  | **Number of Surgeries** | -0.02 (0.2) | 0 (0, 0) |
|  | **Services provided by health professionals** | 0.11 (4.31) | 0 (0, 0) |
|  | **Diagnostic tests** | **-5.06 (15.94)** | -6.5 (-15.5, 3) |
|  | **Laboratory tests** | -2.14 (10.26) | -0.5 (-8.5, 3) |
|  | **Medical equipment** | 0 (0.71) | 0 (0, 0) |
|  | Other | -0.06 (1.14) | 0 (0, 0) |

Note: OD = Organic Disease; D/FSD = Difficult Functional Somatic Disorder; O/FSD = Functional Somatic Disorder with Organic Disease; S/FSD = Simple Functional Somatic Disorder; SED = Stress Exacerbated Disease.

**Supplementary Table S5. Changes in total healthcare costs for the study population and DCG**

|  |  | **Delta** | |  |
| --- | --- | --- | --- | --- |
|  | **Variable** | **Mean (SD)** | **Median (IQR)** | **P-Value** |
| **Total population (N=685)** | Total cost | **-1366.76 (39,315.13)** | **-568.78 (-197,002.5, 647,233)** | **<.0001** |
|  | Hospitalizations | -1722.82 (33688.67) | 0 (-2425.61, 0) | <.0001 |
|  | Surgical Procedures | 216.91 (11926.72) | 0 (0, 0) | 0.4593 |
|  | Specialist Consultations | 721.81 (6363.51) | 213 (-124, 676) | <.0001 |
|  | Services provided by health professionals | 38.35 (1385.89) | 0 (-40, 152) | 0.0362 |
|  | Diagnostic tests | -495.45 (2403.95) | -237 (-1632, 520) | <.0001 |
|  | Laboratory tests | -49.49 (483.76) | -7 (-121, 68) | 0.0005 |
|  | Medications | 19.46 (5275.82) | 18.89 (-153.26, 335.75) | 0.0001 |
|  | Medical equipment | 48.38 (4849.9) | 0 (0, 0) | 0.8054 |
|  | Other | -143.89 (1782.6) | 0 (-689, 5) | <.0001 |
| **OD (N=207)** | Total cost | **266.75 (65269.42)** | **-271.8 (197002.5, 647233)** | **0.114** |
|  | Hospitalizations | 182.64 (57865.87) | 0 (-7331.47, 0) | 0.0003 |
|  | Surgical Procedures | -173.24 (17706.08) | 0 (0, 0) | 0.5476 |
|  | Specialist Consultations | 1131.78 (10604.12) | 228 (-124, 686) | <.0001 |
|  | Services provided by health professionals | 51.1 (1966.17) | 0 (-118, 137) | 0.7826 |
|  | Diagnostic tests | -234.32 (2488.57) | -163 (-1684, 787) | 0.0321 |
|  | Laboratory tests | -92.61 (526.04) | -4 (-133, 69) | 0.0438 |
|  | Medications | -199.8 (8882.02) | 127.24 (-107.17, 641.5) | <.0001 |
|  | Medical equipment | -252.01 (6030.57) | 0 (0, 0) | 0.8207 |
|  | Other | -146.81 (2482.15) | 0 (-701, 1) | 0.0087 |
| **D/FSD (N=110)** | Total cost | **-2129.31 (18292.47)** | **99.89 (-161712.83, 29004.31)** | **0.686** |
|  | Hospitalizations | -2552.19 (17072.92) | 0 (0, 0) | 0.029 |
|  | Surgical Procedures | 277.88 (3492.74) | 0 (0, 0) | 0.969 |
|  | Specialist Consultations | 586.92 (1333.51) | 391 (-63.52278, 994.19484) | <.0001 |
|  | Services provided by health professionals | 99.59 (1540.02) | 0 (-8, 228) | 0.1222 |
|  | Diagnostic tests | -497.09 (1948.81) | -34.5 (-1408, 482) | 0.0404 |
|  | Laboratory tests | -68.1 (486.57) | -15.5 (-95, 37) | 0.0457 |
|  | Medications | 13.91 (781.52) | 0 (-169.91, 184.84) | 0.7257 |
|  | Medical equipment | 0 (0) | 0 (0, 0) | --- |
|  | Other | -57.13 (1950.37) | 0 (-700, 0) | 0.0267 |
| **O/FSD (N=125)** | Total cost | **-4074.1 (30550.46)** | **-2022.43 (-92569.45, 203538.8)** | **0.0008** |
|  | Hospitalizations | **-4922.91 (18735.02)** | **0 (-4960.7, 0)** | **<.0001** |
|  | Surgical Procedures | 461.05 (12893.74) | 0 (0, 0) | 0.7987 |
|  | Specialist Consultations | **1016.41 (5733.63)** | **153 (-325, 756)** | **0.0094** |
|  | Services provided by health professionals | -92.32 (1240.61) | 0 (-182, 284) | 0.7664 |
|  | Diagnostic tests | **-1012.62 (2875.92)** | **-511 (-2600, 608)** | **0.0002** |
|  | Laboratory tests | -27.26 (656.92) | -15 (-140, 59) | 0.0693 |
|  | Medications | -5.49 (1622.5) | 16.93 (-187.21, 427.67) | 0.3622 |
|  | Medical equipment | 683.17 (8292.54) | 0 (0, 0) | 0.8984 |
|  | Other | -174.11 (1296.52) | 0 (-699, 684) | 0.366 |
| **S/FSD (N=143)** | Total cost | **-1096.17 (12369.25)** | **-773.64 (-58024, 57634.5)** | **0.0073** |
|  | Hospitalizations | **-1765.49 (7451.34)** | **0 (0, 0)** | **0.0007** |
|  | Surgical Procedures | 741.57 (8600.23) | 0 (0, 0) | 0.4543 |
|  | Specialist Consultations | 310.34 (868.55) | 142 (-112, 519) | <.0001 |
|  | Services provided by health professionals | 97.85 (611.37) | 0 (0, 132) | 0.024 |
|  | Diagnostic tests | **-473.36 (2531.58)** | **-262 (-1482, 383)** | **0.0031** |
|  | Laboratory tests | -28.15 (309.37) | -8 (-120, 75) | 0.1919 |
|  | Medications | 256.54 (2610.27) | -6.2 (-143.34, 176.14) | 0.6766 |
|  | Medical equipment | -0.13 (12.17) | 0 (0, 0) | 0.875 |
|  | Other | -235.35 (1241.43) | 0 (-689, 0) | 0.022 |
| **SED (N=100)** | Total cost | **-912.08 (6489.72)** | **-362.32 (-22070.47, 42534.74)** | **0.018** |
|  | Hospitalizations | **-693.71 (3354.18)** | **0 (0, 0)** | **0.0361** |
|  | Surgical Procedures | -171.63 (1716.25) | 0 (0, 0) | >0.999 |
|  | Specialist Consultations | 241.67 (789.15) | 160.5 (-97.5, 426.5) | 0.0009 |
|  | Services provided by health professionals | 22.87 (345.89) | 0 (0, 5.5) | 0.381 |
|  | Diagnostic tests | **-419.34 (1679.44)** | **-276.5 (-1477.5, 425.5)** | **0.011** |
|  | Laboratory tests | 1.94 (307.3) | 0 (-128, 91) | 0.4687 |
|  | Medications | 171.59 (3769) | 25.585 (-160.67, 240.33) | 0.2719 |
|  | Medical equipment | -0.73 (13.46) | 0 (0, 0) | >0.999 |
|  | Other | -64.75 (776.28) | 0 (0, 0) | 0.4762 |

Note: OD = Organic Disease; D/FSD = Difficult Functional Somatic Disorder; O/FSD = Functional Somatic Disorder with Organic Disease; S/FSD = Simple Functional Somatic Disorder; SED = Stress Exacerbated Disease.
